# Rheumatic Heart Disease: Molecules Involved in Valve Tissue Inflammation Leading to the Autoimmune Process and Anti-*S. pyogenes* Vaccine

**DOI:** 10.3389/fimmu.2013.00352

**Published:** 2013-10-30

**Authors:** Luiza Guilherme, Jorge Kalil

**Affiliations:** ^1^Heart Institute (InCor), School of Medicine, University of São Paulo, São Paulo, Brazil; ^2^Immunology Investigation Institute, National Institute for Science and Technology, University of São Paulo, São Paulo, Brazil; ^3^Clinical Immunology and Allergy Division, School of Medicine, University of São Paulo, São Paulo, Brazil

**Keywords:** *S. pyogenes*, genes, adhesion molecules, chemokines, Th1 and Th17 cytokines, T and B cells, valve proteins, anti-*S. pyogenes* vaccine

## Abstract

The major events leading to both rheumatic fever (RF) and rheumatic heart disease (RHD) are reviewed. Several genes are involved in the development of RF and RHD. The inflammatory process that results from *S. pyogenes* infection involves the activation of several molecules such as VCAM and ICAM, which play a role in the migration of leukocytes to the heart, particularly to the valves. Specific chemokines, such as CXCL3/MIP1α as well as CCL1/I-309 and CXCL9/Mig, attract T cells to the myocardium and valves, respectively. The autoimmune reactions are mediated by both the B- and T-cell responses that begin at the periphery, followed by the migration of T cell clones to the heart and the infiltration of heart lesions in RHD patients. These cells recognize streptococcal antigens and human-tissue proteins. Molecular mimicry between streptococcal M protein and human proteins has been proposed as the triggering factor leading to autoimmunity in RF and RHD. The production of cytokines from peripheral and heart-infiltrating mononuclear cells suggests that T helper 1 and Th17 cytokines are the mediators of RHD heart lesions. The low numbers of IL-4 producing cells in the valvular tissue might contribute to the maintenance and progression of the valve lesions. The identification of a vaccine epitope opens a perspective of development of an effective and safe vaccine to prevent *S. pyogenes* infections, consequently RF and RHD.

## Introduction

Rheumatic fever (RF) and its major sequelae rheumatic heart disease (RHD) are autoimmune diseases that arise following infection of the throat by *S. pyogenes* in children and young individuals (3–19 years old) who present genetic components that confer susceptibility to the disease.

The disease still remains a major cause of cardiovascular disability in school children and young individuals, and it represents a high burden for public health in the developing world. The incidence of this disease in the so-called “hotspots” ranges from 20 to 51 per 100,000 habitants, causing ∼500,000 deaths each year (1). In Brazil, the number of beta hemolytic streptococcus throat infections is ∼10 million cases/year, leading to 30,000 new cases of RF, of which ∼15,000 cases develop RHD (2).

The aim of this review is to explore the role of several genes in the control of *S. pyogenes* infection and the associated autoimmune reactions, as well as to depict the molecular mechanisms leading to these autoimmune reactions.

### Genetic background

As RF and RHD are post-infectious diseases that involve an inflammatory reaction in addition to T and B cells, several genes are involved in the predisposition and manifestation of the disease. Table 1 summarizes the genes involved in RF/RHD development and their role.

**Table 1:** **Genes of genetic susceptibility of RF and RHD**.

Genetic markers	Role
MBL; TLR2; FCN2; FCγRIIa	Innate immunity
	Inadequate immune response against *S. pyogenes*
HLA class II genes (DR and DQ, several alleles)	Adaptive immune response T cell antigen presentation and immune response
TNF-α, ILRA, TGF-β, IL-10	Both innate immunity/adaptive immune response
	Mediators of inflammatory reactions

### Genes related to the innate immune response

The first line of host defense against a pathogen, *S. pyogenes* in the cases of RF and RHD, involves several molecules that bind to specific pathogen-associated molecular patterns (PAMPs) through specific molecules in the host, defined as pattern recognition receptors (PRRs). These PRRs can be soluble in human serum, or they can be cell-associated, and they are described below.

Toll-like receptors (TLRs) are sensors of foreign microbial products that initiate host defense responses in multicellular organisms. The genotype 753Arg/Gln of *TLR*2 gene resulting from the replacement of arginine with glutamine at codon 753 was more frequently present in a Turkish ARF cohort compared with controls (3).

Mannan-binding lectin (MBL) is a phase I inflammatory protein encoded by different variants of the promoter and exon 1 regions of the *MBL2* gene. The A and O alleles code for high and low production of MBL, respectively. Interestingly, RHD patients with mitral stenosis (MS) displayed an association with the A allele, while the majority of RHD patients with aortic regurgitation (AR) presented the O allele. The amount of MBL in the sera of RF and RHD patients presented high and low serum levels of MBL, respectively (4, 5). These results suggest that the *MBL2* gene could play a role in the development of valvular stenosis or regurgitation.

Ficolins trigger the innate immune response by either binding to collectin cellular receptors or initiating the complement lectin pathway. There have been three ficolin genes identified in humans with different functions, sequences, and specificity. Polymorphisms at −986, −602, and −4 within the promoter region of ficolin 2 (*FCN2*) are associated with the serum levels of this protein. In Brazilian chronic RHD patients, the haplotype G/G/A (−986/−602/−4) was more frequent than in controls and correlated with low levels of this protein, leading to a prolonged time of infection or to repeated streptococcal infections (6).

### Genes related to the adaptive immune response

The susceptibility of developing RF/RHD was first associated with alleles of the HLA class II genes (*DRB1*, *DQB*, and *DQA*). Among the DRB1 alleles, HLA-DR2, DR3, DR4, DR7 were the most frequently associated with the disease, with HLA-DR7 being the most consistently associated HLA allele found in Brazilian, Turkish, Egyptian, and Latvian RF/RHD patients [reviewed in Ref. (7)]. The role of the HLA molecules encoded by these genes is to present antigens to the T cell receptor (TCR), thus activating the adaptive immune response.

### Genes related to both the innate and the adaptive immune response

The *TNF*-α gene has an inflammatory role and is located on the same chromosome as the HLA class II genes. The polymorphism of a SNP at the promoter region of TNFA-308G/A was associated with the susceptibility of patients from Mexico, Turkey, Brazil, and Egypt to RHD (8–11).

IL-1α and IL-1β are cytokines that have been implicated in the inflammatory reactions and are encoded by *IL-1Ra* gene. The most common alleles are 1 and 2, which encode antagonists of IL-1α and IL-1β. The absence or misrepresentation of both alleles results in a strong inflammatory response. Studies in Brazilian RHD patients with severe carditis showed low frequencies of allele 1, suggesting the absence of inflammatory control (12). Some studies showed that alleles of the *TGFβ1* gene were risk factors for the development of valvular RHD lesions (13, 14) as this gene codes for an inflammatory protein secreted by many cell types including macrophages. Thus inflammatory stimuli that activate macrophages enhance the release of active TGF-β.

## Heart Valve Chronic Inflammation

The healing process of rheumatic carditis results in varying degrees of fibrosis and valve damage. The Aschoff body is considered the hallmark of the disease and consists of a granulomatous nodule usually located in the connective tissue around small vessels. This structure promotes the inflammatory process as the mediator of rheumatic heart lesions. Several inflammatory cells, such as neutrophils, macrophages, and T and B lymphocytes, infiltrate both the myocardium and the valves. These cells enter through the myocardium and the valves upon the upregulation of expression of the adhesion molecules. Cunningham’s group showed that streptococcal heart-tissue cross-reactive antibodies increased the amount of VCAM-1 on the valvular endothelial surface, leading to myocarditis and valvulitis (15, 16). Recently, we verified that ICAM, another integrin, was also upregulated, in addition to P-selectin and several chemokines and their receptors. Among the chemokines, CCL3/MIP1α gene expression was up regulated in the myocardium, while CCL1/I-309 and CXCL9/Mig were highly expressed in the valvular tissue of RHD patients (17). An *in vitro* assay demonstrated that valvular lesions infiltrating T cells migrated mainly toward a CXCL9/Mig gradient, suggesting that specific chemokines can mediated both the CD4^+^ and CD8^+^ T cell recruitment to the site of inflammation in the heart (17).

Cytokines are important secondary signals following an infection because they trigger effective immune responses in most individuals, and they most likely cause deleterious responses in patients with autoimmune disease. Cytokines generally act locally.

In RHD, in both the myocardium and valvular tissue, a large number of infiltrating mononuclear cells secreting IFNγ and TNFα inflammatory cytokines were found. Interestingly, only small numbers of IL-4 producing cells were found in the valves, while several cells producing IL-10 were observed. These data strongly indicated that the low numbers of IL-4 producing cells may contribute to the progression of valve lesions in RHD (18). Recently, we identified large numbers of IL-17 and IL-23-producing cells in the valves; IL-17 and IL-23 are a Th17 subset cytokines that are also frequently involved in the development of autoimmune diseases (19). All of the events currently known to be involved in inflammation and infiltration of the heart tissue by T cells are summarized in Table 2.

**Table 2:** **Molecules involved with heart-tissue cellular infiltration**.

Adhesion molecules (VCAM, ICAM) P-selectin and integrins are overexpressed in the heart-tissue and facilitates the cellular infiltration, valve scarring
Specific chemokines such as CCL3/MIP1α gene expression up regulated in the myocardium and CCL1/I-309 and CXCL9/Mig in the valves recruited auto-reactive T cells
High numbers of TNF-α and IFN-γ, IL-17, and IL-23 secreting mononuclear cells are mediators of myocardium and valvular inflammation and drive the autoimmune response
Low numbers of mononuclear IL-4 secreting cells in the valves probably lead to permanent and progressive valvular damage
Infiltrating T cells are predominantly CD4^+^ (∼80%)
Antigen-driven oligoclonal T cells are expanded in the myocardium and valves and recognize streptococcal M peptides and heart-tissue proteins by cross reactivity
The degeneracy of TCR and the epitope spreading mechanism allowed the recognition of several streptococcal and human proteins with some degree of homology (sequences or conformational or chemical properties)

### Autoimmune reactivity

The existence of similar or identical antigens in microbes (virus, bacteria, and other pathogens) and their hosts enable the microbe to evade the host immune response. The mechanism known as “molecular mimicry,” by which self antigens are recognized after an infection by cross reactivity, was introduced by Damian (20).

The presence of heart-reactive antibodies was described more than 50 years ago in sera from animals immunized with streptococcal cell wall products and in sera from acute RF and RHD patients.

Using immunofluorescence techniques, Kaplan found immunoglobulins and complement bound to the myocardium of acute RF patients (21). Studies conducted by Zabriskie et al. gave support to the hypothesis that RF has an autoimmune origin by describing the presence of antibodies that were cross reactive with streptococcal membrane antigens in acute RF sera (22).

Many studies have focused on identifying the cross-reactive streptococcal epitope recognized by antibodies in sera from both animals and humans (23). Identification of the amino acid sequences of the N-terminal portion of the M protein in the 1980s led to the discovery of cross-reactive epitopes. The molecular mimicry between group A streptococcal proteins and several human-tissue proteins leads to the autoimmune reactions in the diverse phenotypes of the disease (24). Sydenham Chorea (SC), one of the major manifestations of the disease affects the central nervous system (CNS), in which lysoganglioside G_M1_ from neuronal cells are the targets of cross-reactive antibodies against *N*-acetyl-β-d-glucosamine, an antigen that is present in the cell wall of *S. pyogenes* (25). In RHD, the valves are severely damaged by both humoral and cellular autoimmune reactions.

The role of the cellular arm of the immune response in RF only began to be investigated 25 years after the description of heart-reactive antibodies in the sera of RF patients. The first studies focused on the reactivity of T cells from the peripheral blood of RF and RHD patients against streptococcal M protein (26, 27). These studies were followed by the description of increased numbers of CD4^+^ cells in the tonsils and peripheral blood of RF patients when compared with healthy subjects (28). The cytotoxic activity of CD8^+^ T cells from normal peripheral blood toward immortalized human heart cells was also described (29). It is now well established that heart-tissue inflammation starts by pericarditis followed by myocarditis and valvulitis, which cause serious damage to the heart valves due to the infiltration of both auto-reactive antibodies and T lymphocytes, leading to the development of valvular lesions and consequently, RHD.

**Figure 1:**
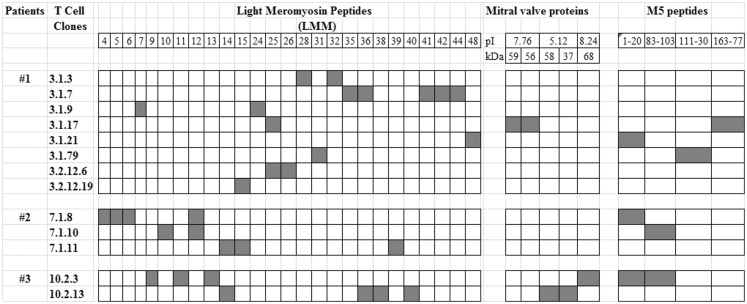
**T cell reactivity against streptococcal M5 protein and heart-tissue proteins**. Valve-derived intralesional T cell clones from three RHD patients who underwent surgery for valve correction were established *in vitro* as previously described (26) and assayed for their reactivity against mitral valve-derived proteins and synthetic peptides of light meromyosin (LMM) and the M5 protein (26, 31). Black box: positive reaction in proliferation assays with a stimulation index (SI) >2.5.

Notably, CD4^+^ cells are predominant in the rheumatic lesions of the heart tissue. An analysis of the reactivity of both heart-tissue infiltrating T cells and peripheral cells against both the N-terminal region M protein-derived peptides and heart-tissue proteins noted three immunodominant regions of the M5 protein (residues 1–25, 81–103, and 163–177) that were cross reactive with both myocardium and valve-derived proteins (26, 27). The recognition of these regions was mainly in the context of the HLA-DR7 molecule, which is one of the most frequent HLA class II alleles associated with the susceptibility of the disease, as mentioned above (27). Among the heart-tissue proteins, cardiac myosin, the most abundant protein in the myocardium, is one of the targets of streptococcal cross-reactive antibodies and heart-tissue-infiltrating T cells. The autoimmune reaction against several synthetic peptides of the beta chain of the cardiac myosin light meromyosin (LMM) region were described by Ellis et al. (30) and Fae et al. (31) and are examples of autoreactivity against the myocardium tissue mediated by both antibodies and T cells. However, it is interesting to note that the permanent rheumatic lesions occur in the valvular tissue, most likely due to the migration of these auto-reactive T cells from the myocardium to the valvular tissue. In the valves, we found several T cell oligoclonal populations defined by the analysis of the TCR (32, 33) that recognized M protein peptides from the N-terminal region and human cardiac myosin beta-chain peptides, as mentioned above, as well as valve tissue-derived proteins (31), as summarized in Figure 1. Among the valve proteins, we identified vimentin and disulfide isomerase ER-60 precursor (PDIA3) protein and a 78-kDa glucose-regulated protein precursor (HSPA5) as targets of the autoimmune reactions (34). It is interesting to note that apparently the recognition by T cells occurs in a cascade of reactivity from the myocardium to the valves.

In summary several cardiac proteins and streptococcal M peptides are recognized by both antibodies and T cells. The cross-reactivity might occur first through mimicry that results in the recognition of other human proteins, especially valve proteins, and eventually through epitope spreading and degeneracy mechanisms that amplified the number of self antigens that are targets of the autoimmune reactions.

### Anti-*S. pyogenes* vaccine development

The epidemiological growth of streptococcal diseases in undeveloped and developing countries has encouraged many groups to study vaccine candidates for preventing Group A Streptococcus (GAS) infections.

There are four anti-GAS vaccine candidates that target the M protein and eight other candidates targeting alternative streptococcal antigens, including group A CHO, C5a peptidase (SCPA), cysteine protease (Spe B), binding proteins similar to fibronectin, opacity factor, lipoproteins, Spes (super antigens), and streptococcal pili (35).

We developed a vaccine epitope (StreptInCor) composed of 55 amino acid residues of the C-terminal portion that is highly conserved among *S. pyogenes*. The StreptInCor epitope is recognized by individuals bearing different HLA class II molecules and could be considered a universal vaccine epitope (36, 37).

Using BALB-c, Swiss, and HLA class II transgenic mice, we evaluated the immune response over an extended period and found that StreptInCor was able to induce a robust immune response in all models (38–40). Vaccinated Swiss mice challenged with a virulent strain of *S. pyogenes* had 87% survival over 30 days. No cross-reaction was observed against cardiac proteins (40). The safety of the vaccine epitope was evaluated by histopathology and no autoimmune or pathological reactions were observed in the heart or other organs (39). Anti-StreptInCor antibodies were able to neutralize/opsonize *S. pyogenes* strains, thus indicating that immunization with StreptInCor is effective against several *S. pyogenes* strains and can prevent infection and subsequent sequelae without causing deleterious reactions (41). These properties are summarized in Table 3. Taking all results into consideration, StreptInCor could be a safe and effective vaccine against streptococcus-induced disease.

**Table 3:** **Properties of “StreptInCor” an anti-*S. pyogenes* candidate vaccine**.

Characteristics	Properties	Reference
M protein C-terminal portion	55 Amino acids residues long	Guilherme et al. (36, 38)
Structure	Alpha helical and beta-sheet conformation, encompasses both T and B epitopes	Guilherme et al. (37)
Experimental assays	Several animal models (BALB/c, C57BL6, Swiss, and HLA class II transgenic mice)	Guilherme et al. (38), Guerino et al. (39), Postol et al. (40), De Amicis Marafigo et al. (41)
Immunogenicity and safety and survival rate	Specific and high titers of opsonic IgG antibodies	Guilherme et al. (38), Guerino et al. (39), Postol et al. (40), De Amicis Marafigo et al. (41)
	Absence of cross reactivity with human heart-tissue proteins	
	Long period of survival after *S. pyogenes* challenge	

## Conclusion

**Figure 2:**
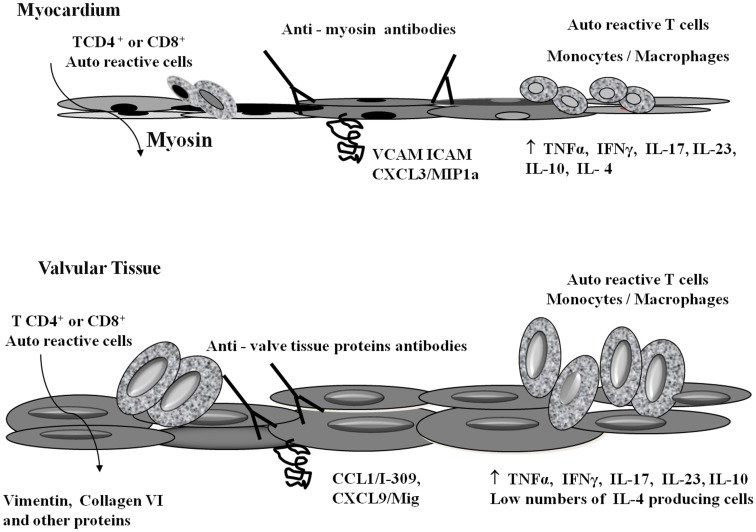
**Schematic representation of heart-tissue infiltration by T cells and autoimmune reactivity**. *S. pyogenes*-reactive T cells infiltrate both the myocardium and valvular tissue through specific integrins (VCAM, ICAM) and chemokines (CCL1/I-309, CXCL3/MIP1α, CXCL9/Mig) (16, 17). Once in the myocardium, T cells recognized cardiac myosin. In the valves, vimentin, collagen VI, and other proteins are the targets of autoimmune reactions. Several inflammatory cytokines are involved in the inflammatory process. The presence of large numbers of regulatory cytokines (IL-4 and IL-10) in the myocardium tissue allowed the cure of myocarditis, while the low numbers of IL-4 producing cells in the valves contributes to the permanent valve damage (18).

The autoimmune process leading to the formation of heart lesions in RHD involves several genes that control both the innate and adaptive immune response. Consequently, several molecules play a role in the different phases of the disease. The molecular mimicry mechanism leads to the recognition of self proteins, mainly heart-tissue proteins, in the case of RHD. The autoimmune reactions are exacerbated by the inflammatory T helper 1 (Th1) and Th17 cytokines. Figure 2 summarizes the events leading to myocarditis and rheumatic valvulitis, and later chronic rheumatic heart disease.

The knowledge acquired by us and others as mentioned through the text, allowed the search of a protective epitope giving a perspective of development of an effective and safe vaccine.

## Conflict of Interest Statement

The authors declare that the research was conducted in the absence of any commercial or financial relationships that could be construed as a potential conflict of interest.

## References

[1] Carapetis JR McDonald M Wilson NJ Acute rheumatic fever. Lancet (2005) 366:155–68 10.1016/S0140-6736(05)66874-2 16005340

[2] Barbosa PJB Müller RE Latado AL Achutti AC Ramos AIO Weksler C Brazilian guidelines for diagnostic, treatment and prevention of rheumatic fever from Cardiology, Pediatric and Rheumatology Brazilian Societies. Braz Arch of Cardiol (2009) 93:1–18

[3] Berdeli A Celik HA Ozyürek R Dogrusoz B Aydin HH TLR-2 gene Arg753Gln polymorphism is strongly associated with acute rheumatic fever in children. J Mol Med (2005) 83:535–41 10.1007/s00109-005-0677-x 15968536

[4] Messias Reason IJ Schafranski MD Jensenius JC Steffensen R The association between mannose-binding lectin gene polymorphism and rheumatic heart disease. Hum Immunol (2006) 67:991–8 10.1016/j.humimm.2006.08.296 17174748

[5] Ramasawmy R Spina G Fae KC Pereira AC Nisihara R Messias Reason IJ Association of mannose-binding lectin gene polymorphism but not of mannose-binding serine protease 2 with chronic severe aortic regurgitation of rheumatic etiology. Clin Vaccine Immunol (2008) 15:932–6 10.1128/CVI.00324-07 18400978 PMC2446618

[6] Messias-Reason IJ Schafranski MD Kremsner PG Kun JF Ficolin 2 (FCN2) functional polymorphisms and the risk of rheumatic fever and rheumatic heart disease. Clin Exp Immunol (2009) 157:395–9 10.1111/j.1365-2249.2009.03975.x 19664148 PMC2745034

[7] Guilherme L Köhler KF Kalil J Rheumatic heart disease: mediation by complex immune events. Adv Clin Chem (2011) 53:31–50 10.1016/B978-0-12-385855-9.00002-3 21404913

[8] Hernández-Pacheco G Flores-Domínguez C Rodríguez-Pérez JM Pérez-Hernández N Fragoso JM Saul A Tumor necrosis factor-alpha promoter polymorphisms in Mexican patients with rheumatic heart disease. J Autoimmun (2003) 21:59–63 10.1016/S0896-8411(03)00079-9 12892736

[9] Sallakci N Akcurin G Köksoy S Kardelen F Uguz A Coskun M TNF-alpha G-308A polymorphism is associated with rheumatic fever and correlates with increased TNF-alpha production. J Autoimmun (2005) 25:150–4 10.1016/j.jaut.2005.05.005 16046099

[10] Ramasawmy R Faé KC Spina G Victora GD Tanaka AC Palácios SA Association of polymorphisms within the promoter region of the tumor necrosis factor-alpha with clinical outcomes of rheumatic fever. Mol Immunol (2007) 44:1873–8 10.1016/j.molimm.2006.10.001 17079017

[11] Settin A Abdel-Hady H El-Baz R Saber I Gene polymorphisms of TNF-alpha(-308), IL-10(-1082), IL-6(-174) and IL-1Ra (VNTR) related to susceptibility and severity of rheumatic heart disease. Pediatr Cardiol (2007) 28:363–71 10.1007/s00246-006-0002-7 17607501

[12] Azevedo PM Bauer R Caparbo Vde F Silva CA Bonfá E Pereira RM Interleukin-1 receptor antagonist gene (IL1RN) polymorphism possibly associated to severity of rheumatic carditis in a Brazilian cohort. Cytokine (2010) 49:109–13 10.1016/j.cyto.2009.09.003 19822442

[13] Kamal H Hussein G Hassoba H Mosaad N Gad A Ismail M Transforming growth factor-beta1 gene C-509T and T869C polymorphisms as possible risk factors in rheumatic heart disease in Egypt. Acta Cardiol (2010) 65:177–83 10.2143/AC.65.2.2047051 20458825

[14] Chou HT Chen CH Tsai CH Tsai FJ Association between transforming growth factor-beta1 gene C-509T and T869C polymorphisms and rheumatic heart disease. Am Heart J (2004) 148:181–6 10.1016/j.ahj.2004.03.032 15215809

[15] Galvin JE Hemric ME Ward K Cunningham M Cytotoxic monoclonal antibody from rheumatic carditis reacts with human endothelium: implications in rheumatic heart disease. J Clin Invest (2000) 106:217–24 10.1172/JCI7132 10903337 PMC314302

[16] Roberts S Kosanke S Terrence Dunn S Jankelow D Duran CM Cunningham MW Pathogenic mechanism in rheumatic carditis: focus on valvular endothelium. J Infect Dis (2001) 183:507–11 10.1086/318076 11133385

[17] Faé KC Palacios SA Nogueira LG Oshiro SE Demarchi LM Bilate AM CXCL9/Mig mediates T cells recruitment to valvular tissue lesions of chronic rheumatic heart disease patients. Inflammation (2013) 36(4):800–11 10.1007/s10753-013-9606-2 23417848 PMC3708284

[18] Guilherme L Cury P Demarchi LM Coelho V Abel L Lopez AP Rheumatic heart disease: proinflammatory cytokines play a role in the progression and maintenance of valvular lesions. Am J Pathol (2004) 165:1583–91 10.1016/S0002-9440(10)63415-3 15509528 PMC1618676

[19] Kikly K Liu L Na S Sedgwick JD The IL-23/Th17axis: therapeutic targets for autoimmune inflammation. Curr Opin Immunol (2006) 18:670–5 10.1016/j.coi.2006.09.008 17010592

[20] Damian RT Molecular mimicry: antigen sharing by parasite and host and its consequences. Am Naturalist (1964) 98:129–49

[21] Kaplan MH Svec KH Immunologic relation of streptococcal and tissue antigens. III. Presence in human sera of streptococcal antibody cross-reactive with heart tissue. association with streptococcal infection, rheumatic fever, and glomerulonephritis. J Exp Med (1964) 119:651–66 10.1084/jem.119.4.651 14151105 PMC2137853

[22] Zabriskie JB Mimetic relationships between group A streptococci and mammalian tissues. Adv Immunol (1967) 7: 147–88 10.1016/S0065-2776(08)60128-5 4868522

[23] Zabriskie JB Hsu KC Seegal BC Heart-reactive antibody associated with rheumatic fever: characterization and diagnostic significance. Clin Exp Immunol (1970) 7:147–59 4920603 PMC1712832

[24] Cunningham MW Pathogenesis of group A streptococcal infections. Clin Microbiol Rev (2000) 13:470–511 10.1128/CMR.13.3.470-511.2000 10885988 PMC88944

[25] Kirvan CA Swedo SE Heuser JS Cunningham MW Mimicry and autoantibody-mediated neuronal cell signaling in Sydenham chorea. Nat Med (2003) 9:914–20 10.1038/nm892 12819778

[26] Guilherme L Cunha-Neto E Coelho V Snitcowsky R Pomerantzeff PM Assis RV Human heart-infiltrating T-cell clones from rheumatic heart disease patients recognized both streptococcal and cardiac proteins. Circulation (1995) 92: 415–20 10.1161/01.CIR.92.3.415 7634457

[27] Guilherme L Oshiro SE Faé KC Cunha-Neto E Renesto G Goldberg AC T cell reactivity against streptococcal antigens in the periphery mirrors reactivity of heart infiltrating T lymphocytes in rheumatic heart disease patients. Infect Immun (2001) 69:5345–535 10.1128/IAI.69.9.5345-5351.2001 11500404 PMC98644

[28] Bhatia R Narula J Reddy KS Koicha M Malaviya AN Pothineni RB Lymphocyte subsets in acute rheumatic fever and rheumatic heart disease. Clin Cardiol (1989) 12:34–8 10.1002/clc.4960120106 2912606

[29] Dale JB Simpson WA Ofek I Beachey E Blastogenic responses of human lymphocytes to structurally defined polypeptide fragments of streptococcal M protein. J Immunol (1981) 126:1499–505 7009743

[30] Ellis NM Li Y Hildebrand W Fischetti VA Cunningham MW T cell mimicry and epitope specificity of cross-reactive T cell clones from rheumatic heart disease. J Immunol (2005) 175:5448–56 16210652 10.4049/jimmunol.175.8.5448

[31] Faé KC da Silva DD Oshiro SE Tanaka AC Pomerantzeff PM Douay C Mimicry in recognition of cardiac myosin peptides by heart-intralesional T cell clones from rheumatic heart disease. J Immunol (2006) 176:5662–70 16622036 10.4049/jimmunol.176.9.5662

[32] Guilherme L Dulphy N Douay C Coelho V Cunha-Neto E Oshiro SE Molecular evidence for antigen-driven immune responses in cardiac lesions of rheumatic heart disease patients. Int Immunol (2000) 12:1063–74 10.1093/intimm/12.7.1063 10882418

[33] Faé K Kalil J Toubert A Guilherme L Heart infiltrating T cell clones from a rheumatic heart disease patient display a common TCR usage and a degenerate antigen recognition pattern. Mol Immunol (2004) 40:1129–35 10.1016/j.molimm.2003.11.007 15036919

[34] Faé KC Oshiro SE Toubert A Charron D Kalil J Guilherme L How an autoimmune reaction triggered by molecular mimicry between streptococcal M protein and cardiac tissue proteins leads to heart lesions in rheumatic heart disease. J Autoimmun (2005) 24:101–9 10.1016/j.jaut.2005.01.007 15829402

[35] Steer AC Batzloff MR Mulholland K Carapetis JR Group A streptococcal vaccines: facts versus fantasy. Curr Opin Infect Dis (2009) 22(6):544–52 10.1097/QCO.0b013e328332bbfe 19797947

[36] Guilherme L Faé KC Higa F Chaves L Oshiro SE Freschi de Barros S Towards a vaccine against rheumatic fever. Clin Dev Immunol (2006) 13(2–4):125–32 10.1080/17402520600877026 17162355 PMC2270766

[37] Guilherme L Postol E Freschi de Barros S Higa F Alencar R Lastre M A vaccine against *S. pyogenes*: design and experimental immune response. Methods (2009) 49(4):316–21 10.1016/j.ymeth.2009.03.024 19409999

[38] Guilherme L Alba MP Ferreira FM Oshiro SE Higa F Patarroyo ME Anti-group A streptococcal vaccine epitope: structure, stability, and its ability to interact with HLA class II molecules. J Biol Chem (2011) 286(9):6989–98 10.1074/jbc.M110.132118 21169359 PMC3044955

[39] Guerino MT Postol E Demarchi LM Martins CO Mundel LR Kalil J HLA class II transgenic mice develop a safe and long lasting immune response against StreptInCor, an anti-group A streptococcus vaccine candidate. Vaccine (2011) 29:8250–6 10.1016/j.vaccine.2011.08.113 21907752

[40] Postol E Alencar R Higa FT Freschi de Barros S Demarchi LMF Kalil J StreptInCor: a candidate vaccine epitope against *S. pyogenes* infections induces protection in outbred mice. Plos One (2013) 8(4):e60969 10.1371/journal.pone.0060969 23593359 PMC3620221

[41] De Amicis Marafigo K Freschi de Barros S Alencar RE Postól E Oliveira Martins C Arcuri HA Analysis of the coverage capacity of the StreptInCor candidate vaccine against *Streptococcus pyogenes*. Vaccine (2013). [Epub ahead of print]. 10.1016/j.vaccine.2013.08.043 23994376

